# Leptin fails to blunt the lipopolysaccharide-induced activation of the hypothalamic–pituitary–adrenal axis in rats

**DOI:** 10.1530/JOE-13-0249

**Published:** 2014-05

**Authors:** Saadia Basharat, Jennifer A Parker, Kevin G Murphy, Stephen R Bloom, Julia C Buckingham, Christopher D John

**Affiliations:** 1 Division of Diabetes, Endocrinology and Metabolism, Department of Investigative Medicine, Imperial College LondonHammersmith Hospital Campus, 6th Floor, Commonwealth Building, Du Cane Road, London, W12 0NN UK; 1 University College London, Institute of Neurology Queen Square House, Queen Square, London, WC1N 3BG UK; 2 Brunel University Wilfred Brown Building, Kingston Lane, Uxbridge, UB8 3PH UK

**Keywords:** stress, leptin, HPA, sepsis

## Abstract

Obesity is a risk factor for sepsis morbidity and mortality, whereas the hypothalamic–pituitary–adrenal (HPA) axis plays a protective role in the body's defence against sepsis. Sepsis induces a profound systemic immune response and cytokines serve as excellent markers for sepsis as they act as mediators of the immune response. Evidence suggests that the adipokine leptin may play a pathogenic role in sepsis. Mouse endotoxaemic models present with elevated leptin levels and exogenously added leptin increased mortality whereas human septic patients have elevated circulating levels of the soluble leptin receptor (Ob-Re). Evidence suggests that leptin can inhibit the regulation of the HPA axis. Thus, leptin may suppress the HPA axis, impairing its protective role in sepsis. We hypothesised that leptin would attenuate the HPA axis response to sepsis. We investigated the direct effects of an i.p. injection of 2 mg/kg leptin on the HPA axis response to intraperitoneally injected 25 μg/kg lipopolysaccharide (LPS) in the male Wistar rat. We found that LPS potently activated the HPA axis, as shown by significantly increased plasma stress hormones, ACTH and corticosterone, and increased plasma interleukin 1β (IL1β) levels, 2 h after administration. Pre-treatment with leptin, 2 h before LPS administration, did not influence the HPA axis response to LPS. In turn, LPS did not affect plasma leptin levels. Our findings suggest that leptin does not influence HPA function or IL1β secretion in a rat model of LPS-induced sepsis, and thus that leptin is unlikely to be involved in the acute-phase endocrine response to bacterial infection in rats.

## Introduction

Sepsis is the result of an over-activation of the innate immune response against bacterial infections (Matot & Sprung 2001) and can result in fever, tachypnea, tachycardia, deregulated organ perfusion, renal failure, hypoxaemia and altered mental state (Bone *et al*. 1989). Biomarkers of sepsis include cytokines such as interleukin 1B (IL1β; Sankar & Webster 2012), as well as other receptor and cell marker biomarkers (Heuer *et al*. 2004). Sepsis progression can result in immune dysfunction (Tschop *et al*. 2010), which is the leading cause for mortality in non-coronary patients in non-Western countries (Arabi *et al*. 2003), comparable with annual death rates from acute myocardial infarction in Western countries. Obesity is a risk factor for sepsis morbidity and mortality. Morbid obesity increases the risk of death by sepsis following major surgery by 50% (Prabhakar *et al*. 2002). It is thought that the pro-inflammatory phenotype that accompanies obesity creates a context in which any additional inflammatory stimulus results in an exaggerated inflammatory response (Vachharajani 2008).

The adipose tissue-derived hormone leptin has a well-characterised role in energy homoeostasis, but has also been suggested to play a pathogenic role in sepsis (Shapiro *et al*. 2010). Mouse endotoxaemia and caecal ligation puncture models of sepsis present with elevated levels of leptin, while exogenous administration of leptin to endotoxaemic mice increases mortality (Shapiro *et al*. 2010). Human septic patients have elevated circulating levels of the soluble leptin receptor (Ob-Re), which correlate with disease severity indices (Shapiro *et al*. 2010). Leptin has been reported to serve as an early biomarker of sepsis in patients, allowing differentiation between patients with systemic inflammatory response syndrome and patients with sepsis (Yousef *et al*. 2010). The hypothalamic–pituitary–adrenal (HPA) axis plays an important protective role in the body's defence against sepsis during a bacterial infection. Bacterial proteins stimulate the release of cytokines, including tumour necrosis factor α (TNFα), IL1 and IL6 (John & Buckingham 2003), which can modulate the HPA axis increasing expression of corticotrophin-releasing hormone (CRH) and arginine vasopressin in the hypothalamus, and adrenocorticotropic hormone (ACTH) in the pituitary gland (John & Buckingham 2003) and increasing circulating glucocorticoid levels (Besedovsky *et al*. 1986). Glucocorticoids themselves act in a negative feedback loop to suppress the HPA axis, leading to a shift from pro-inflammatory immune responses to anti-inflammatory immune responses (Elenkov & Chrousos 1999). There is evidence to suggest that leptin has an inhibitory role in the regulation of the HPA axis (Maffei *et al*. 1995). Leptin administration blunts the restraint stress-induced activation of the HPA axis in mice (Heiman *et al*. 1997) and can directly inhibit ACTH-stimulated cortisol/corticosterone release from human and rat adrenal cells *in vitro* (Gaillard *et al*. 2000). Thus, the possible pathogenic role for leptin in sepsis may be linked to leptin-induced suppression of the HPA axis impairing the protective role of the HPA axis in sepsis.

We hypothesised that leptin attenuates the HPA axis response to sepsis. We investigated the direct effects of leptin on the HPA axis response to acute lipopolysaccharide (LPS) administration in rats, a model of the neuroimmunological changes observed in sepsis in both rodents and humans. Acute LPS administration increases circulating levels of the cytokines IL1β, IL6 and TNFα and up-regulates the HPA axis (Besedovsky *et al*. 1986, Rivier *et al*. 1989, Takao *et al*. 1993, Csontos *et al*. 2010). If leptin acts to exacerbate the pathophysiological response to LPS, it would suggest a possible role for anti-leptin therapy in high-risk septic patients.

## Materials and methods

### Animals

Male Wistar rats (specifically pathogen free; Charles River, Margate, UK) weighing 170–210 g and at 7 weeks of age were maintained in groups of four under controlled temperature (21±1 °C) and light (12 h light:12 h darkness cycle; lights on at 0700 h) with access to food (RM1 diet, Special Diet Services Ltd, Witham, Essex, UK) and water and were allowed to feed *ad libitum*. They were handled daily for 2 weeks before the study. All animal procedures conducted were approved by the British Home Office under the Animals (Scientific Procedures) Act 1986.

### Materials

LPS from *Escherichia coli* was supplied by Sigma–Aldrich Ltd. Recombinant rat leptin was supplied by R&D Systems (Abingdon, UK).

### Study design

Leptin has been shown to significantly reduce basal plasma corticosterone levels, 3–4 h after administration (Clark *et al*. 2008). Plasma stress hormone levels peak at 1–2 h after i.p. LPS administration, at doses ranging from 10.5 to 250 μg/kg (Takemura *et al*. 1997, Gaillard *et al*. 2000, Tolchard *et al*. 2009), to levels comparable with those observed in septic rats (Carlson *et al*. 2007). Rats (*n*=10/group) were administered with either 100 μl vehicle (saline) or 2 mg/kg recombinant rat leptin intraperitoneally, a dose previously shown to suppress the HPA axis response to acute restraint stress (Heiman *et al*. 1997) and to activate regions of the brain involved in the regulation of energy balance (Elmquist *et al*. 1997). After 2 h, rats received a second i.p. injection of either 100 μl vehicle (saline) or 25 μg/kg LPS, a dose that induces a significant, albeit reversible, increase in HPA activity (Huang *et al*. 1998). All rats were killed by decapitation 2 h after the second injection and trunk blood was immediately collected into plastic potassium-EDTA tubes (1.6 mg EDTA/mm of blood). Plasma was separated by centrifugation for 10 min at 2000 r.p.m. (Boeco Centrifuge S-8, Boeco, Hamburg, Germany) and stored at −70 °C for measurement of ACTH, corticosterone, leptin and IL1β concentrations.

### Plasma hormone measurements

Plasma ACTH levels were measured by IRMA (DiaSorin, Stillwater, MN, USA); intra-assay variation was 3.5–4.8% and inter-assay variation was 3.2–5.7%. Plasma corticosterone levels were determined by enzyme immunoassay (Cayman Chemical Company, Cambridge, UK); intra-assay variation was 8% and inter-assay variation was 9.8%. Plasma leptin levels were determined by ELISA (Crystal Chem, Inc., Downers Grove, IL, USA); both intra-assay and inter-assay variations were ≤10%. Plasma IL1β levels were determined by ELISA R&D Systems; intra-assay variation was 3.9–8.8% and inter-assay variation was 4.1–5.7%. All assays were performed according to the manufacturers' instructions.

### Statistical analysis

All data are presented as mean±s.e.m. Groups were compared using one-way ANOVA followed by Tukey's *post-hoc* test with GraphPad Prism version 5 (GraphPad Software, Inc., La Jolla, CA, USA). In all cases, *P*<0.05 was considered statistically significant.

## Results

### Effect of leptin on the HPA axis response to LPS

**Figure 1:**
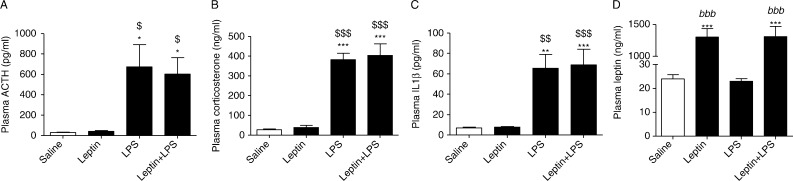
The effect of leptin on LPS-stimulated increases in ACTH, corticosterone and IL1β concentrations. Male Wistar rats were intraperitoneally injected with 100 μl saline or 2 mg/kg leptin 2 h before being intraperitoneally injected with 100 μl saline or 25 μg/kg LPS. Rats were decapitated 2 h after LPS injection and plasma (A) ACTH, (B) corticosterone, (C) IL1β and (D) leptin concentrations were measured. Data are presented as mean±s.e.m. one-way ANOVA. *P<0.05 vs saline; **P<0.01 vs saline; ***P<0.001 vs saline; ^$^ *P*<0.05 vs leptin; ^$$^ *P*<0.01 vs leptin; ^$$$^ *P*<0.001 vs leptin; ^bbb^ *P*<0.001 vs LPS and *n*=8–10.

At 2 h after administration, 25 μg/kg LPS significantly increased plasma ACTH (674±217 pg/ml) and corticosterone (381.8±32.6 ng/ml) levels (Fig. 1A and B). Pre-treatment with 2 mg/kg leptin, 2 h before LPS administration, did not significantly influence the HPA axis response to LPS (ACTH, 608±160 pg/ml; corticosterone, 403.3±59.2 ng/ml) (Fig. 1A and B). LPS did not affect plasma leptin (23±1.09 ng/ml) levels (Fig. 1D) but did significantly increase plasma IL1β (65.5±13.6 pg/ml) levels (Fig. 1C) compared with untreated controls. Leptin had no effect on basal (7.77±0.714 pg/ml) or LPS-stimulated (68.8±15.5 pg/ml) plasma IL1β concentrations (Fig. 1C). As expected, plasma leptin levels were significantly higher in rats that received leptin compared with rats that did not receive leptin (Fig. 1D).

## Discussion

We aimed to investigate the effect of leptin on the neuroendocrine response to LPS in the male Wistar rat. Our studies suggest that peripheral leptin administration does not affect the HPA axis or the cytokine response to LPS-induced endotoxaemia.

Based on the evidence in the literature that LPS activates the HPA axis at doses ranging from 10.5 to 250 μg/kg in rats, we initially carried out a dose–response study investigating the effects of various doses of LPS (10, 25, 100, 250 μg/kg) on HPA axis activation (S Basharat, KG Murphy, SR Bloom, JC Buckingham & CD John, 2012, unpublished observations). We found 25 μg/kg LPS to be the lowest dose that significantly elevated plasma ACTH and corticosterone levels at the pertinent time points in our model (*n*=10). Others have previously shown that our chosen dose of LPS induces a strong but reversible stress response, as demonstrated by the inhibition of 25 μg/kg LPS-induced fever by systemic administration of α-MSH in rats (Huang *et al*. 1998). Plasma ACTH and corticosterone levels measured 2 h after i.p. LPS administration within the study by Huang and colleagues were similar to those achieved in our study. We measured IL1β as an early marker of sepsis in our model as it is one of the cytokines most strongly associated with sepsis and, in both human and experimental septic models, has been shown to act as an ‘initiator’ cytokine that stimulates a cytokine cascade, ultimately driving many of the physiological changes seen in sepsis (Blackwell & Christman 1996). The plasma IL1β levels achieved in our LPS-treated rats are similar to levels observed in septic humans (Kurt *et al*. 2007, Sankar & Webster 2012) and fall into the range of IL1β levels observed in animal models of sepsis (Rodríguez-Wilhelmi *et al*. 2002). Our chosen dose of leptin was the same as that used in the study carried out by Heiman *et al*. (1997), which demonstrates that leptin can blunt restraint stress-induced HPA activation in mice 3–4 h after its administration. Our study was designed to use the same time points but with a different stressor (LPS), given that interpreting the contradictory literature regarding the effects of leptin on the HPA axis is difficult due to the variations in the time points investigated, mode of plasma and tissue collection and animal model used. Clark *et al*. (2008) also utilised the same leptin dose and time point as our study. In contrast to our finding, they found that leptin suppressed baseline corticosterone levels, perhaps because their rats had higher basal corticosterone levels (200 ng/ml compared with 25 ng/ml in our study (Clark *et al*. 2008)), which may perhaps reflect differences in housing or strain. It may be difficult to detectably suppress corticosterone concentrations below relatively low basal levels. In our study, 2 mg/kg leptin induced circulating plasma leptin levels well above those seen in obese rats (Beck & Richy 2009).

It is possible that investigating the effects of different doses of LPS and leptin, and/or measuring HPA axis activation at different time points might reveal effects of leptin that we do not detect in this study. Conflicting reports suggest that leptin can stimulate or suppress the HPA axis in different contexts (Maffei *et al*. 1995, Heiman *et al*. 1997, Nishiyama *et al*. 1999, Jethwa *et al*. 2006) and suggest that animal model, type of stressor and route of leptin administration can greatly influence study outcome. Evidence for the suppressive effects of leptin on the HPA axis includes the ability of leptin to blunt the stress-induced activation of the HPA axis in mice (Heiman *et al*. 1997), inhibit CRH release from stimulated *ex vivo *rat hypothalamic explants (Heiman *et al*. 1997) and attenuate ACTH-stimulated cortisol release from adrenal cells *in vitro* (Bornstein *et al*. 1997, Gaillard *et al*. 2000). The presence of the leptin receptor (LEPR) in ACTH-producing cells of the anterior pituitary gland (Zamorano *et al*. 1997) suggests that they are targets of leptin signalling, though incubation of primary rat pituitary cells with leptin does not influence CRH-induced ACTH release (Heiman *et al*. 1997). Peripheral leptin administration to fasted mice attenuates the fasting-induced activation of the HPA axis as demonstrated by normalised plasma ACTH and corticosterone levels comparable to those seen in fed mice (Ahima *et al*. 1996), while systemic administration of leptin in male Sprague–Dawley rats decreases baseline plasma corticosterone levels (Clark *et al*. 2008).

However, under specific conditions, leptin can stimulate the HPA axis. I.c.v. administration of leptin has been shown to increase *Crh* and CRH receptor (*Crhr2*) mRNA expression in the rat hypothalamic paraventricular nucleus and ventromedial nucleus (Nishiyama *et al*. 1999). Leptin also stimulates CRH release from hypothalamic explants (Costa *et al*. 1997, Raber *et al*. 1997, Jethwa *et al*. 2006) and Jethwa *et al*. (2006) reported an acute stimulatory effect of peripheral leptin on the HPA axis in rats.

Such variable findings stem from the absence of uniformly designed and directly comparable *in vivo* leptin-stress experiments. Most studies have focused on the ability of leptin to influence the HPA response to mild stressors, whereas our study has focused on the effects of leptin on the HPA response to a powerful immunological stressor. It is interesting to note that while different stressor types, e.g. head trauma (Ott *et al*. 1987), stroke (Kang *et al*. 2008), autoimmune diseases (Hu *et al*. 1993) and psychological stress (Connor & Leonard 1998), ultimately activate the HPA axis via a convergence at the hypothalamus to precipitate the CRH–ACTH–glucocorticoid cascade, there is surprisingly little overlap in the sets of genes within the brain that are induced/repressed by severe immunological stress compared with mild psychological stressors such as restraint stress (Reyes *et al*. 2003). Furthermore, in addition to the convergent effect at the hypothalamus, LPS can act directly at the pituitary gland to stimulate the inter-pituitary release of IL6, which subsequently up-regulates ACTH secretion (Gloddek *et al*. 2001, Watanobe & Yoneda 2003), and also within the adrenal cortex (Andreis *et al*. 1991, Pournajafi Nazarloo *et al*. 2003, Mohn *et al*. 2011). Leptin could feasibly reduce adrenal gland sensitivity to ACTH stimulation, though correlation analyses of plasma ACTH and corticosterone concentrations in our animals indicate a moderate positive correlation, suggesting that there is not a marked reduction in ACTH sensitivity at the adrenal gland. Therefore, the fact that LPS i) induces a different activational response within the brain compared with mild stressors and ii) activates the HPA axis at multiple levels may provide an explanation as to why leptin failed to attenuate the LPS-induced HPA response.

In conclusion, our findings show that an acute dose of 2 mg/kg leptin fails to influence HPA function or IL1β secretion, 4 h after injection, in a rat model of acute 25 μg/kg LPS-induced sepsis. This suggests that leptin may not to be involved in the acute-phase response to bacterial infection in rats.

## Author contribution statement

S B, C D J and K G M designed the study, S B was responsible for the animal management, S B and J A P administered the injections, S B conducted the plasma assays and S B, C D J and K G M wrote the paper.
